# Social Structure Predicts Genital Morphology in African Mole-Rats

**DOI:** 10.1371/journal.pone.0007477

**Published:** 2009-10-15

**Authors:** Marianne L. Seney, Diane A. Kelly, Bruce D. Goldman, Radim Šumbera, Nancy G. Forger

**Affiliations:** 1 Center for Neuroendocrine Studies and Department of Psychology, University of Massachusetts, Amherst, Massachusetts, United States of America; 2 Department of Geological Sciences, Amherst College, Amherst, Massachusetts, United States of America; 3 Department of Ecology and Evolutionary Biology, University of Connecticut, Storrs, Connecticut, United States of America; 4 Department of Zoology, University of South Bohemia, České Budejovice, Czech Republic; University of Arizona, United States of America

## Abstract

**Background:**

African mole-rats (Bathyergidae, Rodentia) exhibit a wide range of social structures, from solitary to eusocial. We previously found a lack of sex differences in the external genitalia and morphology of the perineal muscles associated with the phallus in the eusocial naked mole-rat. This was quite surprising, as the external genitalia and perineal muscles are sexually dimorphic in all other mammals examined. We hypothesized that the lack of sex differences in naked mole-rats might be related to their unusual social structure.

**Methodology/Principal Findings:**

We compared the genitalia and perineal muscles in three African mole-rat species: the naked mole-rat, the solitary silvery mole-rat, and the Damaraland mole-rat, a species considered to be eusocial, but with less reproductive skew than naked mole-rats. Our findings support a relationship between social structure, mating system, and sexual differentiation. Naked mole-rats lack sex differences in genitalia and perineal morphology, silvery mole-rats exhibit sex differences, and Damaraland mole-rats are intermediate.

**Conclusions/Significance:**

The lack of sex differences in naked mole-rats is not an attribute of all African mole-rats, but appears to have evolved in relation to their unusual social structure and reproductive biology.

## Introduction

Sex differences in the external genitalia and in muscles associated with the genitalia are nearly universal among mammals. Males commonly have a larger phallus (used here to denote either the penis or clitoris) and/or anogenital distance than females [1]. Striated muscles that attach to the phallus, such as the bulbocavernosus (BC; also known as bulbospongiosus), ischiocavernosus (IC), and levator ani (LA; sometimes referred to as the dorsal BC; [2], [3]), are also much larger in males; in fact, these muscles are absent or vestigial in females of many rodents [4], [5]. The morphology and function of the striated perineal muscles have been best studied in rats. The BC and LA attach exclusively to the bulb of the penis, whereas the IC attaches at its distal end to the corpora cavernosa and at its proximal end to the ischium [6], [7], [8]. These muscles are rhythmically active during penile erection and ejaculation in rats, dogs, and humans [6], [8], [9], [10], and surgical removal severely impairs fertility in male rats [8].

We recently reported a surprising lack of sex differences in the external genitalia and perineal muscles of naked mole-rats (*Heterocephalus glaber*) [11], [12]. Naked mole-rats are small rodents native to Africa that exhibit the most extreme form of cooperative breeding in a mammal [13]. They are, in fact, considered to be eusocial, a term originally used to describe social insects [14]. Most naked mole-rat colonies are comprised of a single breeding female (the queen), one to three breeding males, and a large number (on average 60–90, but in some cases over 200) of non-reproductive “subordinates,” who assist in foraging, colony defense, maintenance of the tunnel system, and caring for the young [14], [15], [16]. Although subordinates can become reproductive if a breeder dies or if a subordinate is removed from a colony and paired with an opposite-sex mate [17], [18], once a breeding animal is established, it is unlikely to be overthrown. It is estimated that, in the wild, the vast majority of individual naked mole-rats never achieve reproductive status [19], [20]. Thus, it is possible that the lack of sex differences in the genitalia and perineal muscles of naked mole-rats might be related to their unusual social structure and breeding system. Alternatively, there might be no such relationship.

The diversity of social organizations within the Bathyergids allows for the opportunity to test the association between sexual dimorphism and degree of sociality, as species range from eusocial to solitary. Recently, Parag and colleagues found that degree of sociality in African mole-rats was correlated with penile ornamentation: solitary species possessed penile spines, while eusocial species lacked spines [21]. Here, we compared the external genitalia and perineal muscles in three species: naked mole-rats, Damaraland mole-rats (*Fukomys damarensis*; formerly *Cryptomys damarensis*; [22]), and silvery mole-rats (*Heliophobius argenteocinereus*). Like naked mole-rats, Damaraland mole-rats are considered eusocial: they live in colonies of about 16 individuals (up to 41 individuals), comprised of a single breeding female (the queen), one to three breeding males, and non-reproductive subordinates [23]. Compared to naked mole-rats, however, Damaraland mole-rats have significantly less reproductive skew (i.e. a higher percentage of individuals will breed; ∼10% in Damaraland mole-rats and 1% in naked mole-rats; [20], [24]). At the other extreme, silvery mole-rats are solitary [13], and all individuals that attain adult body size may potentially reproduce.

We reasoned that if the absence of sex differences in naked mole-rats is due to their social and breeding system, we might observe sex differences in solitary silvery mole-rats and Damaraland mole-rats might be intermediate. Our results support a relationship between sex differences and social structure.

## Materials and Methods

### Animals

**Figure 1:**
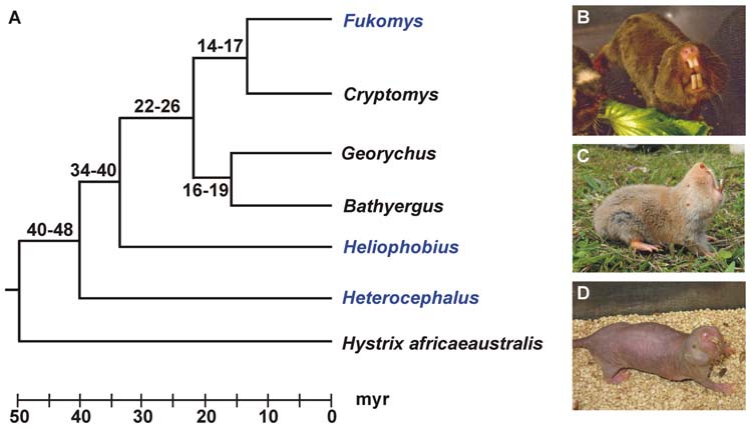
Phylogeny of African mole-rats. (A) Phylogenetic relationships and approximate divergence times of African mole-rat species and one outgroup species (*Hystrix africaeaustralis*). Divergence times assume a molecular clock calibration of between 40 and 48 million years (myr) for the basal node within the family. The three species compared in the current study are members of the genera highlighted in blue. Adapted from [29]. (B–D) Photographs of a (B) Damaraland mole-rat (*Fukomys damarensis*), (C) silvery mole-rat (*Heliophobius argenteocinereus*), and (D) naked mole-rat (*Heterocephalus glaber*). Photograph credits: (B) and (C) by Sharry Goldman; (D) by Radim Šumbera.

The phylogenetic relationships between the three African mole-rats used in this study are shown in Figure 1.

#### Naked mole-rats

Colonies were maintained at the University of Connecticut, Storrs in polypropylene tubs (with Plexiglas™ lids) containing corncob bedding and connected by lengths of acrylic tubing. Animals were descended from individuals captured in Kenya, as described previously [11] and were fed *ad libitum* on a diet consisting of sweet potato supplemented with apples, carrots, squash, and oatmeal. Rooms were maintained on a 12∶12 light/dark photoperiod at 29–30°C. All animals were of adult body size (22–67 g) and ranged in age from 1 and 12 years.

#### Damaraland mole-rats

Colonies were maintained at Storrs in tubs as described for naked mole-rats and fed *ad libitum* on a diet consisting of sweet potato supplemented with other vegetables, cereal, and cornmeal. Rooms were maintained on a 16∶8 light/dark photoperiod at 22–26°C. All animals were adults between 1 and 8 years of age and 98–254 g body mass.

#### Silvery mole-rats

Animals were collected in the wild in 2000 in Malawi at two sites: Blantyre-Limbe and Mulanje, Chipoka village (for details on particular locales, see [25]). With the exception of one male who was collected in September, all animals were collected between late April 20 and June 1, which corresponds to the “cold-dry” season and the period during which most mating occurs [26]. Although ages of the individuals were not known, all were of adult body size (135–276 g) and appearance, and thus were presumably at least six months old.

#### Tissue collection and processing

For naked and Damaraland mole-rats, perineums were dissected out of freshly killed specimens, immersion fixed in formalin, transferred to Bouin's solution for 2 weeks, and stored in 70% ethanol. Silvery mole-rats killed in the field were initially preserved in 70% ethanol. Upon arrival at the University of Massachusetts, tissues were switched to Bouin's solution for 2 weeks, and stored in 70% ethanol prior to processing. For microscopic analyses of perineal structures, blocks of tissue were embedded in paraffin, sectioned at 10 µm, and mounted on gelatin-subbed slides. Sections were stained with Gomori's trichrome to enhance the detection of muscle striations.

#### Ethics Statement

All procedures were approved by the Institutional Animal Care and Use Committee at the University of Connecticut.

### Morphology of the external genitalia and perineal muscles

Anogenital distance and phallus length were measured in all three species using dial calipers and genitalia were digitally photographed (Canon Powershot A650 IS attached to a dissecting microscope). Anogenital distance was defined as the distance between the center of the anal opening to the center of the base of the phallus. Phallus length was defined as the distance between the base and tip of the phallus. All measures were performed on live animals, except for phallus length in silvery mole-rats which was measured following fixation. Tissues from a small number of animals from each species (n = 2 per sex) underwent gross dissection to examine the overall morphology of the phallus, perineal muscles, and supporting structures; drawings were made of these dissections.

We previously quantified perineal muscle volumes in naked mole-rats [11], [12]. In the present study, we measured the IC and UM muscles in Damaraland and silvery mole-rats. Our previous studies found no sex differences in muscle size of the IC or UM in subordinate or breeder naked mole-rats; we quantified the IC and UM muscles in subordinate Damaraland mole-rats here. Each muscle was traced in sections spaced 400 µm apart through the perineum. Volume was determined by summing cross-sectional areas and multiplying by the distance between traced sections, as previously [12]. Because the IC was similar in morphology and attachment sites in all three species, we also compared the absolute IC volume and IC volume corrected for body weight in males across species.

### Data analysis

For naked and Damaraland mole-rats, anogenital distance and phallus length were compared by 2-way ANOVA (sex-by-status). Silvery mole-rats do not have animals of different social status; we compared anogenital distance and phallus length in male and female silvery mole-rats by independent 2-tailed t-tests. Sex differences in volume of the perineal muscles within a species were tested using independent t-tests. IC muscle volumes were compared across species using a 1-way ANOVA, followed by planned comparisons using Fisher's Least Significant Difference. Means were reported ± SEM and p<0.05 was considered statistically significant.

## Results

### External genitalia

#### Naked mole-rats

**Figure 2:**
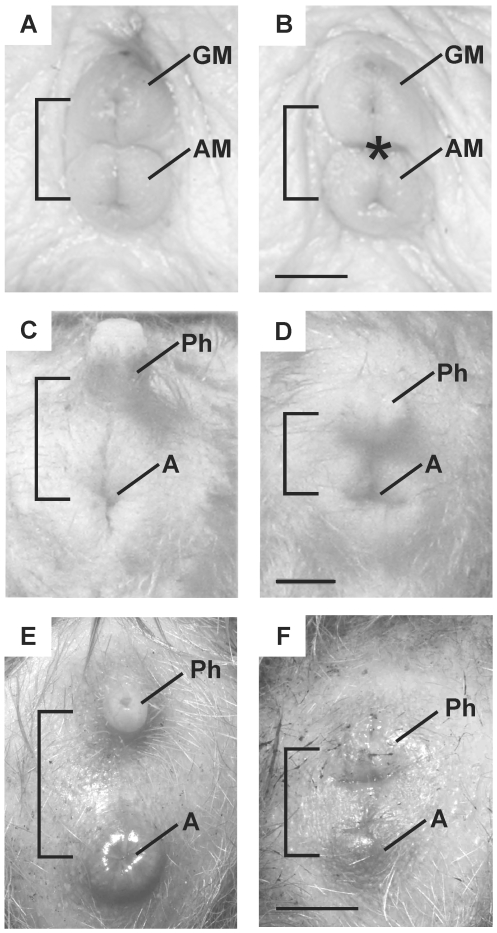
External genitalia in three African mole-rat species. Naked mole rat male (A) and female (B); Damaraland mole-rat male (C) and female (D); Silvery mole-rat male (E) female (F). Brackets indicate anogenital distance. Asterisk in B indicates site of the imperforate vagina. Abbreviations: A, anus; AM, anal mound; GM, genital mound; Ph, phallus. Scale bars: 3 mm for A–D; 2 mm for E and F.

As reported previously, the external genitalia are remarkably monomorphic in naked mole-rats [11], [14]. The testes of both subordinate and breeding males are non-scrotal and the external penis consists of a small genital mound just superior to and abutting the anal mound (Fig. 2A). Female subordinates have an imperforate vagina, which opens only if a female becomes a queen [14] and the external genitalia of females are nearly identical to those of males (Fig. 2B). We found no effect of sex (p>0.25) or status (p>0.45), and no sex-by-status interaction (p>0.95) on phallus length (Table 1). We did find a small (0.54 mm), but consistent, effect of sex on anogenital distance (p<0.002), although in contrast to most rodents, anogenital distance was actually greater in females than in males. We attribute the slightly increased anogenital distance in naked mole-rat females to the presence of the vagina (or future vaginal opening) that increases the space between the rectum and urethra (Figure 2B). We also found an effect of status (p<0.01), with breeders having larger anogenital distances than subordinates, with no sex-by-status interaction (p>0.75).

**Table 1:** Mean (± SEM) anogenital distance (AGD) and external phallus length in naked, Damaraland, and silvery mole-rats.

	AGD (mm)	Phallus length (mm)^1^
**Naked mole-rats**		
**♀** subordinates (n = 5)	2.78±0.08	1.80±0.10
**♀** breeders (n = 6)	3.28±0.15	1.88±0.10
**♂** subordinates (n = 4)	2.28±0.17	1.93±0.10
**♂** breeders (n = 6)	2.70±0.17	2.02±0.13
**Damaraland mole-rats**		
**♀** subordinates (n = 5)	5.30±0.21	3.14±0.15
**♀** breeders (n = 5)	5.20±0.12	2.82±0.15
**♂** subordinates (n = 5)	6.66±0.19	5.32±0.33
**♂** breeders (n = 5)	6.80±0.36	5.54±0.55
**Silvery mole-rats**		
**♀** (n = 11)	5.46±0.17	2.10±0.15
**♂** (n = 8)	6.28±0.19	3.35±0.26

1 All phallus length measures were performed on live specimens except in silvery mole-rats, where measures were post-fixation. Thus, shrinkage due to fixation may have reduced these measures in silvery mole-rats, but presumably did so to a similar extent in males and females.

#### Damaraland mole-rats

Similar to naked mole-rats, the testes of Damaraland mole-rats are non-scrotal; the vagina is imperforate in subordinates and open in breeders. However, in contrast to naked mole-rats, males and females are easily distinguished by simple visual inspection (Figure 2C, D) and phallus length and anogenital distance are sexually dimorphic in the traditional mammalian pattern (Table 1). Males had greater phallus lengths than females (p<0.001), with no effect of status (p>0.8) and no sex-by-status interaction (p>0.4). Males also had greater anogenital distances, with no effect of status (p>0.9) and no sex-by-status interaction (p>0.6) on this measure.

#### Silvery mole-rats

The testes of silvery mole-rat males are non-scrotal, but the external genitalia are readily distinguishable between the sexes (Figure 2E, F). Males have significantly longer phalluses and anogenital distances than females (p<0.01 for both comparisons; Table 1). The vagina was perforate in all specimens examined.

### Internal penis morphology

**Figure 3:**
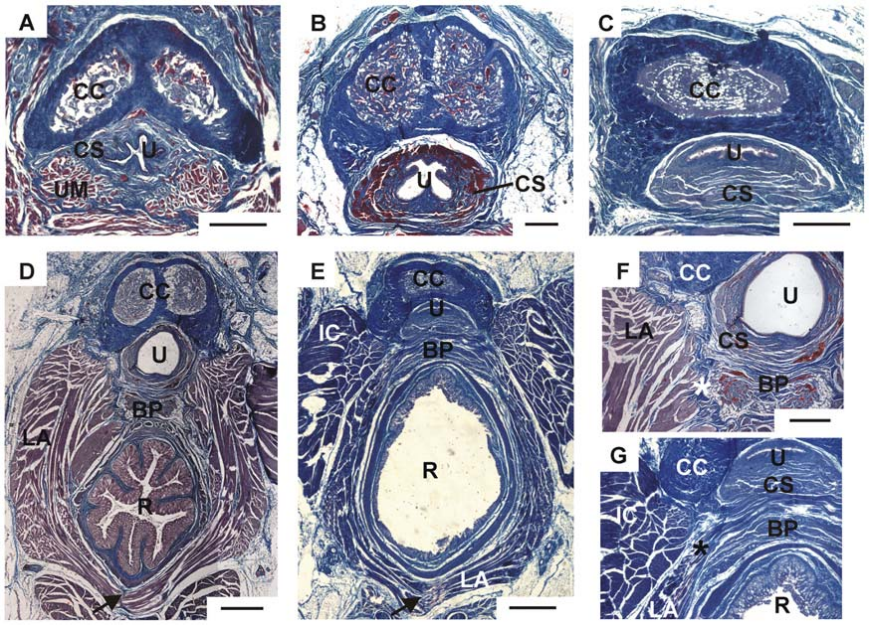
Comparison of penile structures in naked, Damaraland, and silvery mole-rats. (A–C) Photomicrographs depicting the structure of the corpora cavernosa and corpus spongiosum in the naked mole-rat, Damaraland mole-rat, and silvery mole-rat, respectively. The urethral muscle is seen at this level in the naked mole-rat (A); this muscle does not extend as far distally in the other two species. (D–E) The bulb of the penis is shown in Damaraland (D) and silvery mole-rats (E), in relation to neighboring structures. (F) Higher power view of the section in (D), showing LA fibers attaching to the bulb of the penis (asterisk) and to the corpus cavernosum. (G) Higher power view of the section in (E), showing LA fibers attaching solely to the bulb of penis (asterisk). Abbreviations: BP, bulb of penis; CC, corpora cavernosa; CS, corpus spongiosum; IC, ischiocavernosus; LA, levator ani; R, rectum; U, urethra. Scale bars in A–C, F, G = 500 µm; D, E = 1 mm.

Most aspects of internal penile morphology were similar across naked, Damaraland, and silvery mole-rats. As in other mammals, the penile shaft contains a pair of hydrostatic erectile elements: the corpus cavernosum penis and the corpus spongiosum penis (Figure 3). The corpus cavernosum is made up of a collagenous membrane that surrounds a mass of highly vascularized tissue. The vascular tissue contains both smooth muscle fibers and strands of more loosely organized collagen. The membrane, called the tunica albuginea, contains highly organized collagen fibers that are crimped and folded in the flaccid penis. The proximal end of the corpus cavernosum splits into a pair of crurae; each crura is attached to the surface of the ipsilateral ischium by a short, broad ligament. The corpus spongiosum is found immediately ventral to the corpus cavernosum, and is held to the midline by a band of collagenous tissue that originates on the ventrolateral surface of the corpus cavernosum and wraps ventrally around the spongiosum. Like the corpus cavernosum, the corpus spongiosum is made up of a collagenous membrane surrounding vascularized tissue, but its membrane is noticeably thinner. The urethra runs through the corpus spongiosum, entering the erectile structure proximally and traveling through the vascularized tissue to exit at the distal end of the penis.

Differences in internal penile morphology among the three species are found primarily in the corpus spongiosum. In silvery and Damaraland mole-rats, the collagenous tissue that holds the spongiosum against the shaft of the corpus cavernosum disappears proximally and the vascular tissue of the corpus spongiosum expands ventrally to form a small penile bulb, as in most mammals (Figure 3). The bulb region is approximately three times larger in the silvery mole-rat than in the Damaraland mole-rat (data not shown). In contrast, naked mole-rats lack a penile bulb. Instead, the band of tissue that holds the corpus spongiosum to the corpus cavernosum persists proximally and encloses the corpus spongiosum as well as the paired urethral muscles (UM; see below).

### Morphology of the perineal muscles

**Figure 4:**
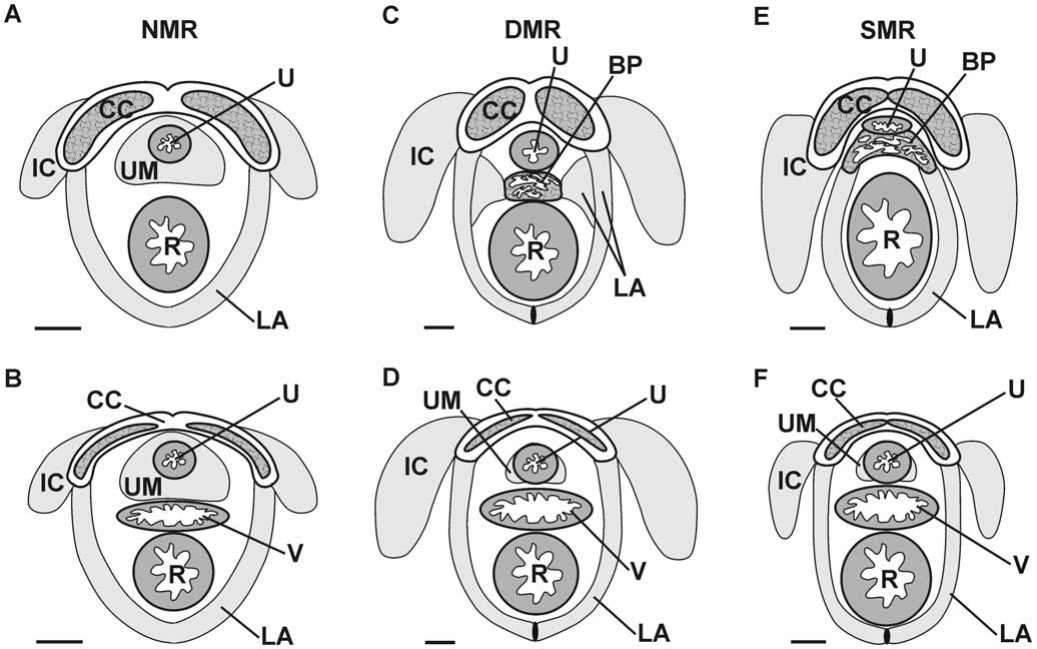
Schematic depictions of sections through the perineal muscles in males and females of three African mole-rat species. Dorsal is down. (A, C, E) Male naked mole-rat, Damaraland mole-rat, and silvery mole-rat, respectively. (B, D, F) Female naked mole-rat, Damaraland mole-rat, and silvery mole-rat, respectively. See text for details. Abbreviations: BP, bulb of penis; CC, corpora cavernosa; IC, ischiocavernosus; LA, levator ani; R, rectum; U, urethra; UM, urethral muscle; V, vagina. Scale bars = 1 mm.

In naked mole-rats, we identified three striated perineal muscles: the IC, LA, and UM (Figure 4; see also [11], [12]). The IC has the same attachment sites as in other rodents (the corpus cavernosum penis or corpus cavernosum clitoridis and the ischium). The BC wraps around the penile bulb in other species and, as mentioned above, naked mole rats lack a bulb of the penis [11], [27]. Thus, no BC is identified in naked mole-rats. However, we do find a striated muscle that encircles the urethra and proximate corpus spongiosum that we refer to as the UM. An LA is identified in naked mole-rats as a band of muscle that loops around the rectum dorsally, with ventral attachment sites to the corpus cavernosum (Figure 4), rather than to the penile bulb. These muscles are compared in the three species, below.

#### Ischiocavernosus

**Figure 5:**
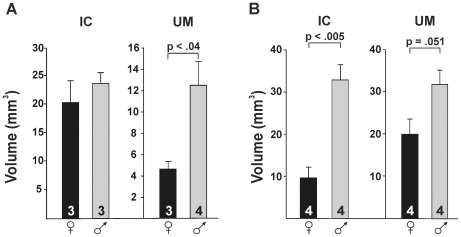
Mean (±SEM) volumes of the ischiocavernosus (IC) and urethral muscle (UM) in (A) Damaraland mole-rats and (B) silvery mole-rats. In Damaraland mole-rats, there is no sex difference in IC volume, but males have a larger UM than females (p<0.04). In silvery mole-rats there is a significant sex difference in IC volume and a nearly significant difference in the same direction for the UM.

Like naked mole-rats, Damaraland and silvery mole-rats also have an IC that attaches to the corpus cavernosum and ischium (Figure 4). This muscle, which is absent or vestigial in female rats or mice [28], is present in both sexes of all three African mole-rat species. However, the degree of sexual dimorphism of the IC varies by species. In naked mole-rats, there is no significant sex difference in IC muscle volume [11], [12] and this is also true in Damaraland mole-rats (p>0.45; Figure 5). However, the IC is significantly larger in male than in female silvery mole-rats (p<0.005; Figure 5).

The volume of the IC also differs across species, both in absolute volume and when corrected for body weight (Table 2). Relative IC volume is one-half the size in naked mole-rat males compared to the other two species (p<0.05 for both comparisons), which do not differ from each other on this measure (p>0.8).

**Table 2:** Mean (± SEM) body weight, absolute IC volume, and IC volume corrected for body weight in male naked, Damaraland, and silvery mole-rats.

	BW (g)	IC volume (mm^3^)	IC volume/BW (mm^3^/g)
**Naked mole-rats (n = 6)**	46±4	3.7±1.0	0.076±0.02
**Damaraland mole-rats (n = 3)**	154±17	23.6±1.8	0.157±0.02
**Silvery mole-rats (n = 4)**	213±25	32.9±3.6	0.164±0.04
**1-way ANOVA**	p<0.001	p<0.001	p<0.04

#### Levator ani

Like naked mole rats, Damaraland and silvery mole-rats have an LA muscle that loops around the rectum. There is a midline raphe joining the left and right halves dorsal to the rectum in Damaraland and silvery mole-rats (lines in LA below the rectum in Figure 4), but no dorsal raphe is seen in naked mole-rats. Some interesting species and sex differences in the ventral attachment sites of this muscle are also seen. In naked mole-rats, the LA attaches to the corpus cavernosum in both males and females. This is also seen for female Damaraland and female silvery mole-rats (Figure 4). In Damaraland mole-rat males, however, attachment sites are mixed: most LA fibers attach to the corpus cavernosum, but others course medially. We find a small bulb of the penis in male Damaraland mole-rats, and, similar to what is seen in mice, rats, and other rodents, the medial LA fibers attach to this structure (Figures 3, 4C). In silvery mole-rat males, we identified a more prominent bulb of the penis (Figures 3, 4E) and the LA attaches solely to this structure in males. Because of the qualitative sex differences in LA attachment sites, we did not attempt to quantify sex differences in LA muscle volume in Damaraland and silvery mole-rats.

#### Bulbocavernosus/urethral muscle

We did not find any muscle directly comparable to the prominent BC of rats, mice, and some other rodents in any of the three African mole-rat species. However, in Damaraland and silvery mole-rats, we find a thin sheet of striated muscle that covers the distal surface of the penile bulb. The left and right halves of this muscle are joined by a raphe. This muscle might be a much reduced BC or a more distal portion of the LA. No such muscle is seen in naked mole-rats.

In naked mole-rats, we previously identified a large UM that surrounds the urethra and proximate corpus spongiosum. This muscle is also present in both Damaraland and silvery mole-rats, although it is not nearly as prominent as in naked mole-rats. A distal extension of the UM seems to be unique to the naked mole-rat: in this species, the muscle runs down the penile shaft and fills the space at the end of the corpus spongiosum which contains the penile bulb in the other species. The UM is not sexually dimorphic in volume in naked mole-rats [11], [12]. However, males have larger UMs than females in Damaraland mole-rats (p<0.04; Figure 5A) and in silvery mole-rats (p = 0.051; Figure 5B), although in the latter case the p-value for the comparison was only marginally significant.

#### Comparison with the mouse

**Figure 6:**
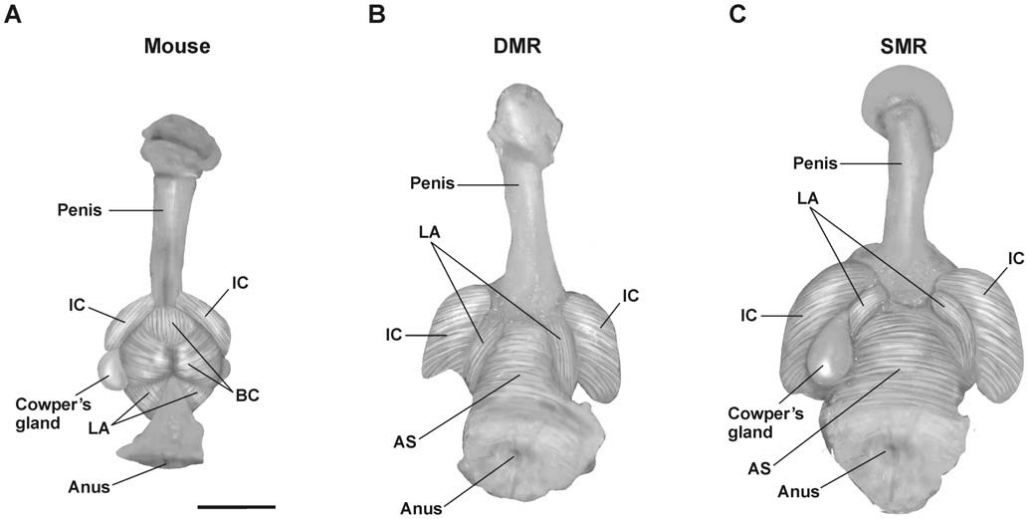
Illustrations of gross dissections of the penis and perineal muscles in a male mouse (A), Damaraland mole-rat (B), and silvery mole-rat (C). Dorsal is down. (A) In the mouse, the LA loops around the rectum and attaches to the base of the penis. Attachment sites of the LA are completely obscured by the bulbous BC, which encircles the ventral LA and also attaches to the penile bulb. A moderately sized IC is seen. (B) Damaraland mole-rats and (C) silvery mole-rats lack a prominent BC, leaving the ventral LA more exposed. Both Damaraland and silvery mole-rats possess large IC muscles and anal sphincters. Cowper's gland has been removed on the right side in (A) and (C). Cowper's gland was smaller and tucked under the LA in Damaraland mole-rats. Abbreviations: AS, anal sphincter; BC, bulbocavernosus; IC, ischiocavernosus; LA, levator ani. Scale bar = 500 µm.

Figure 6 presents illustrations of gross dissections of the perineums of male Damaraland and silvery mole-rats, with a male mouse shown for comparison. The African mole-rats have much larger IC muscles and anal sphincters than do mice, but lack the prominent, bulbous BC seen in other male rodents. They instead possess a tube-like muscle surrounding the urethra (UM; not evident in the gross dissections).

## Discussion

The range of social systems within the Bathyergids affords us the opportunity to relate the degree of sexual differentiation to sociality. Species within this family are all endemic to Africa and are subterranean, but social systems range from eusocial to solitary. Three genera were sampled here. Of these, the eusocial genus *Heterocephalus* is phylogenetically the oldest, followed by *Heliophobius*; species in the genus *Fukomys* have evolved more recently [29], [30]. It is not known whether the common ancestor for all three genera was solitary or eusocial (see [13], [31]). With either scenario, sociality has evolved (or has been lost) more than once within the Bathyergidae [13]. The fact that naked and Damaraland mole-rats seem to have separately evolved eusociality makes the comparisons in the current study more dependent upon social structure than relatedness.

**Figure 7:**
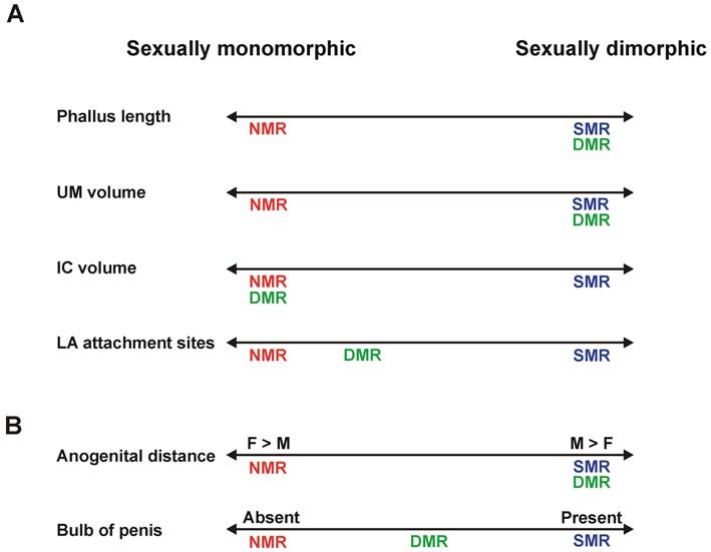
Schematic depicting the pattern of results for traits examined in this study. (A) Phallus length, UM volume, IC volume, and LA attachment sites were all sexually dimorphic in silvery mole-rats (SMR). These traits were not significantly dimorphic in naked mole-rats (NMR). In Damaraland mole-rats (DMR), an intermediate pattern was seen: phallus length and UM size were dimorphic, but IC volume was monomorphic. LA attachment sites were mixed in male DMRs, but more similar to that of NMR. (B) Anogenital distance is sexually dimorphic in all African mole-rats examined, but followed an opposite pattern in naked mole-rats (F>M) compared to Damaraland or silvery mole-rats (M>F). The bulb of penis is present in silvery mole-rats, while absent in naked mole-rats. Damaraland mole-rats have a small bulb of penis.

Here, we found numerous differences in genital morphology among three species within the African mole-rat family, and several of these differences correlate with social structure. Figure 7 presents a schematic summary of our main findings. On at least four measures where eusocial naked mole-rats are sexually monomorphic, solitary silvery mole-rats exhibit sexual dimorphism. Similar to males of other rodent species, male silvery mole-rats have a greater phallus length and anogenital distance than do females, and there are significant sex differences in perineal muscle volumes (Figure 7). None of these features are seen in naked mole-rats. Damaraland mole-rats resemble silvery mole-rats with respect to sex differences in phallus length, anogenital distance, and UM volume. However, as in naked mole-rats, the IC muscle was not significantly dimorphic. Our sample sizes were small, so it is possible that subtle sex differences in IC size would be detected in naked mole-rats, Damaraland mole-rats, or both, with the inclusion of more animals. Nonetheless, it is clear that a sex difference of the magnitude seen in rats and mice (which is absolute, as females lack the muscle) or silvery mole-rats (a greater than 3-fold sex difference) is not present. For two other features (presence or absence of the penile bulb and LA attachment sites) Damaraland mole-rats were intermediate (Figure 7, and see below for further discussion).

A reduction in sex differences in the genitalia has been described in other species, most often in association with female masculinization. For example, striking virilization of the genitalia is observed in female moles of the genus Talpa, the ring-tailed lemur and, most notably, the spotted hyena [32], [33], [34]. Interestingly, female dominance and the absence or reversal of the usual male advantage in body size are also seen in the lemurs and spotted hyenas, suggesting that masculinization of females and, hence a reduction in sex differences, may be common in female-dominated societies. However, the lack of sex differences in the genitalia of naked mole-rats does not appear to be a case of female masculinization. A more apt characterization may be feminization of males because the external penis is very small, a penile bulb is absent, and anogenital distance is nearly zero (the phallic mound abuts the anal mound).

In other mammals, sex differences in the genitalia and the perineal muscles are dependent on developmental exposure to androgens [4], [35]. No developmental studies have been performed in any African mole-rat species to determine, for example, when the gonads differentiate or whether there is a perinatal testosterone surge in males, as is seen in other mammals. Given the long gestation period of each of the species studied here (roughly 70, 85, and 95 days for naked mole-rats, Damaraland mole-rats and silvery mole-rats, respectively [13], [26]), we predict a prenatal period of sexual differentiation. In support of this suggestion, we recently treated Damaraland mole-rats with testosterone during approximately the last third of gestation and found that the external genitalia and UM were masculinized in the female offspring of androgenized mothers (unpublished observations). The absence of sex differences in naked mole-rats suggests that either sex differences in androgen exposure are minimal, or that some tissues that are androgen sensitive in most species are not responsive to androgens prenatally in this species.

We suspect that an important factor driving the evolution of a reduction of sex differences in naked mole-rats may be the extreme reproductive skew in this species. The genitalia of Damaraland mole-rats are clearly dimorphic and those of naked mole-rats are monomorphic, despite the fact that both species are eusocial. The breeding systems of these species differ in some important ways, however. Compared to Damaraland mole-rats, naked mole-rats exhibit larger colony size and a greater degree of reproductive suppression of subordinates [13], [36]. When given a choice, naked mole-rats may prefer outbreeding [37], but DNA fingerprinting suggests that colonies in the wild are highly inbred [38]. In contrast, Damaraland mole-rats exhibit strict incest avoidance [39] and have a higher likelihood of dispersal. These factors contribute to the more extreme reproductive skew in naked mole-rat colonies, where only about 1% of animals are thought to ever engage in reproduction [20], [24].

Sperm competition is also thought to be an important factor driving the evolution phallus size and other sex differences [40]. Silvery mole-rats may exhibit multiple-male paternity [41], so strong erections and sperm competition may be relevant to reproductive success in this species. The breeding female in naked mole-rat colonies is thought to choose the male(s) with which she will mate [24], [42] and once a male becomes a breeder, he is likely a breeder for life. Moreover, males in a naked mole-rat colony are all closely related to each other [38]. Thus, there is little occasion for competition between males. A similar argument has been put forth to explain the tiny penis of the male gorilla, which measures only about 3 centimeters in the erect state [40], [43]. Gorillas live in stable social groups with a single dominant male. Estrous females solicit copulations and do so exclusively from the alpha male resulting in the near absence of sperm competition in this species [40], [43].

There were no notable differences in corpus cavernosum morphology among the three species, which is not surprising, as the anatomy of this structure is conserved across mammals. We did, however, find some interesting variation in the corpus spongiosum among the three African mole-rat species. In most mammals, the base of the corpus spongiosum expands to form a mass of spongy vascular tissue called the bulb of the penis [44]; in rats, the bulb is covered with a fibrous layer to which the BC and LA attach [2]. Here we report that Damaraland and silvery mole-rats possess a bulb of the penis. The bulb is more substantial in the silvery mole-rat than it is in the Damaraland mole-rat, even though these species are similar in body size. In our previous studies [11], [12], we were unable to identify a bulb of the penis in naked mole-rats, similar to the observations of Hill and colleagues [27]. The vascular tissue of the corpus spongiosum in naked mole-rats is instead surrounded by a distal extension of the UM which fills the space that contains the penile bulb in silvery and Damaraland mole-rats. The function of the UM is unknown, but it is possible that UM contractions during ejaculation could create pressure waves in the corpus spongiosum vascular space to move semen through the urethra, thereby compensating for the absent BC.

Thus, presence of the penile bulb also correlates with reproductive skew: no visible bulb in naked mole-rats, a small bulb in Damaraland mole-rats, and a larger bulb in solitary silvery mole-rats. LA attachment sites in the three mole-rat species follow a similar pattern. In rats, the LA attaches to the bulb of the penis in males, while the tiny remnant of an LA that is present in females has no well-defined attachment point [2], [4]. We previously reported that both male and female naked mole-rats have a substantial LA that attaches exclusively to the corpus cavernosum [11]. We also find a substantial LA in Damaraland and silvery mole-rats of both sexes. In females of both of these species, the LA attaches to the corpus cavernosum, as in naked mole-rats. However, the LA of silvery mole-rat males attaches exclusively to the bulb of the penis. LA attachment sites are intermediate in male Damaraland mole-rats, with most LA fibers attaching to the corpus cavernosum, but about a third projecting to the bulb of the penis. Thus, attachment sites are completely sexually dimorphic for silvery mole-rats, monomorphic in naked mole-rats, and mixed in Damaraland mole-rats. Čihák and colleagues suggest that the LA develops from the same blastema as the IC [4]. This may suggest an explanation for why the LA attaches to the corpus cavernosum (as the IC does) in naked and Damaraland mole-rat males and females and silvery mole-rat females.

Some features previously described in naked mole-rats apply to all three species we examined, and therefore are unlikely to be related to social or reproductive system. For example, although silvery and Damaraland mole-rats exhibit sex differences in anogenital distance, these sex differences are not as great as those seen in mice and rats [1]. In addition, all of the African mole-rats we studied lack the prominent BC muscle found in males of many other mammals. The BC rhythmically contracts during erection and ejaculation [8], [9], [10] and these contractions increase pressure in the corpus spongiosum [45]. After surgical excision of the BC and LA, male rats can copulate, but rarely succeed in impregnating a female [8]. Contractions of the BC also are important for the production of penile cups in rats, which serve to dislodge seminal plugs left by previous males and tightly lodge the new seminal plug to the cervix after ejaculation [6], [8]. Based on observations of others (N. Bennett, personal communication), the social mole-rats do not form copulatory plugs; whether copulatory plugs are found in solitary species such as the silvery mole-rat is not known.

Although all of the African mole-rats studied lacked a definitive BC, both males and females of all three species possessed a UM and IC, and the IC of Damaraland and silvery mole-rats is strikingly large. In males, contractions of the IC primarily affect the corpus cavernosum penis, creating suprasystolic pressures inside the vascular space that increase penile rigidity [45]. IC contractions in rats also change the angle of the penis relative to the rest of the body, producing dorsiflexions (“flips”) of the glans penis; surgical excision of the IC prevents “flips” and subsequent penetration of the vagina [8]. It is possible that the large IC in Damaraland and silvery mole-rats produces glans movements inside the female during copulation. Although female rats and mice lack any IC muscle, an IC is present in human females and numerous other female mammals and may serve to maintain an erect clitoris [5].

Darwin suggested that some sexual dimorphisms evolve and are accentuated in relation to competition between members of one sex for mating opportunities [46]. We propose that pronounced sex differences in the genitalia and perineal muscles may not be required in eusocial species in which most individuals will never engage in attempts to breed and where there is little or no male-male competition. A similar argument may be made for dimorphisms in other features. Several authors have argued for a connection between social system and degree of sexual dimorphism in overall body size in mammals or ornamentation in birds (e.g., [47]). In addition, we recently noted that naked mole-rats also lack sex differences in regions of the brain that are sexually dimorphic in other mammals [48]. Similarly, in voles, the sexually dimorphic nucleus of the preoptic area and anteroventral periventricular nucleus of the hypothalamus are less sexually dimorphic in monogamous than in polygamous species [49]. Thus, it is possible that sexual differentiation of the brain (and, by extension, behavior) is related to social structure in a manner similar to what is found for the genitalia.
